# Mechanism of Mycotoxin Contamination of Medicinal Herbs

**DOI:** 10.3390/toxins17030139

**Published:** 2025-03-14

**Authors:** Abdelrahman Elamin, Shohei Sakuda

**Affiliations:** Department of Biosciences, Teikyo University, 1-1 Toyosatodai, Utsunomiya City 320-8551, Tochigi Prefecture, Japan; a.elamin@nasu.bio.teikyo-u.ac.jp

**Keywords:** jujube fruit, lotus seed, licorice, aflatoxins, ochratoxins, hilar region, HPLC, SEM, mechanism, susceptibility

## Abstract

Mycotoxin contamination in medicinal plants can lead to toxicity, reduced therapeutic efficacy, and economic losses. This contamination has emerged as a significant issue, drawing attention from researchers and research centers worldwide. Over recent decades, numerous analytical studies have addressed mycotoxin contamination in these herbs, evaluating various methods to determine their presence quantitatively and qualitatively. While several reviews have summarized these studies, they often overlook a comprehensive exploration of the mechanisms and influencing factors of mycotoxin contamination in medicinal herbs. Therefore, this review aims to delve into the mechanisms of aflatoxin and ochratoxin contamination in some of the most widespread medicinal herbs, including jujube fruits, lotus seeds, and licorice roots. The factors influencing these mechanisms were also examined, including the physical composition and maturity stages of the herbs. This review concluded that aflatoxin and ochratoxin A contamination of medicinal herbs involves complex interactions between the herbs’ natural defenses, fungal pathogenicity, chemical composition, physical characteristics, and individual plant differences at various maturity stages. Understanding these mechanisms of contamination, and their association with maturity, nutrient profile, and physical development, advances our comprehension of mycotoxin contamination in medicinal herbs.

## 1. Mycotoxin Contamination of Medicinal Herbs

Since ancient times, plants have been utilized in medicine, with approximately ten percent of all vascular plants serving as medicinal plants [1,2]. Various parts of plants, such as the leaf, stem, bark, and root, are used to prevent or alleviate symptoms and revert abnormalities to normal [3]. These plants serve as primary healthcare resources in developing countries and are commonly part of the self-medication trend in developed nations [4]. The use of medicinal herbs is strongly influenced by familiarity with these herbs, the social impact of herbalists, and the perceived usefulness of the herbs [5]. However, despite their therapeutic effects, medicinal herbs can cause adverse effects such as hepatotoxicity, cardiovascular toxicity, and central nervous system alterations, primarily due to the toxic effects of active plant compounds or contamination during cultivation and manufacturing processes [4].

Medicinal herbs are highly susceptible to toxigenic fungal infections and mycotoxin contamination, which can occur both during the pre- and postharvest stages [6]. These plants often grow in poor environmental conditions and are produced through traditional, small-scale, open workshops and scattered planting models. The lack of uniform standards or efficient supervision regarding processing, storage, and transportation contributes to significant contamination by mycotoxigenic fungi, leading to the accumulation of mycotoxins. Regulating mycotoxin levels in herbal preparations is a growing concern [7,8,9].

**Figure 1:**
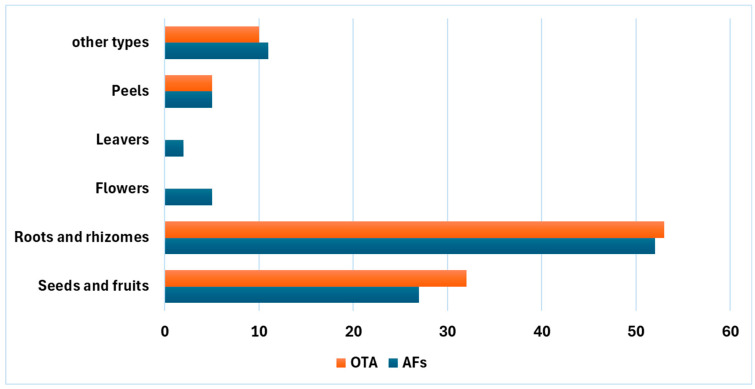
The susceptibility (%) of the different parts of medicinal plants to AFs and OTA contamination [6].

Mycotoxins are toxic secondary metabolites produced by fungi such as *Aspergillus*, *Penicillium*, *Fusarium*, *Claviceps*, and *Alternaria*, with approximately 400 different types identified. The mycotoxins most harmful to medicinal herbs include aflatoxins (AFs), ochratoxins (OTs), fumonisins, zearalenone, and deoxynivalenol [8]. These naturally occurring compounds are found in a wide range of agricultural products and medicinal herbs worldwide [10]. Recent reports on mycotoxin contamination in medicinal herbs and related products have shown that AFs and OTs are the most prevalent contaminants [11,12]. Roots and seeds are particularly susceptible to contamination with OTs and AFs (Figure 1) [6], likely due to the direct contact of roots with soil fungi and the rich starch, protein, and fat contents of seeds [9]. Table 1 provides a summary of reports on AF and OT contamination in various medicinal herbs, including jujube, lotus, and licorice, which will be studied in detail in the subsequent sections.

The risk assessment of AFB_1_ in herbal medicines and plant food supplements in the Malaysian market containing *Salvia officinalis* L., *Centella asiatica* (L.) Urb., *Piper nigrum* L., *Trachyspermum ammi* L., *Phoenix dactylifera* L., *Nigella sativa* L., *Crocus sativus* L., *Zingiber officinale* Roscoe, *Punica granatum* L., and others was evaluated [13]. Out of 31 samples analyzed using the ELISA method, 25 (80.6%) were found to be contaminated with AFB_1_ at levels ranging from 0.275 to 13.941 μg/kg. Notably, four samples (12.9%) exhibited high levels of contamination. The AF levels in two different herbal medicines (for malaria and typhoid; each prepared with water and alcohol) from Itoku Market in Ogun State, Nigeria, were detected [14]. *A. flavus* strains were isolated from the typhoid herbs (bark of *Enantia chlorantha* Oliv., *Sarcocephalus latifolium* (Sm.) E.A. Bruce, *Garcinia kola* Heckel, and *Cocos nucifera* L.) prepared with water. AF quantification was conducted on the herbal samples using HPLC-FLD. Typhoid herbs prepared with water showed high AF detection limits of 7600.0 μg/kg. AFs and OTs were identified in 48 contaminated samples of 13 different medicinal herbs obtained from the herbal market in China [9]. HPLC-FLD analysis indicated that 70.8% of the herbs had slight contamination with AFs (<5.0 μg/kg). *Codonopsis radix* samples contained OTA (360.0–515.0 μg/kg), and *Scutellariae radix* (the dried roots of *Scutellaria baicalensis* Georgi) samples contained OTA (49.0–231.0 μg/kg). The predominant mycoflora included *Aspergillus* spp. (26.1%) and *Penicillium* spp. (24.6%). Multiplex PCR analysis showed that three *A. flavus* strains harbored AF biosynthesis genes. One *A. flavus* strain isolated from *Amomi fructus* produced AFB_1_ and AFB_2_. The levels of AFs and OTA in 36 South African medicinal plants, including *Harmbstaedti aodorata* (Burch.) T. Cooke, *Vachellia karroo* (Hayne) Banfi & Galasso, and *Cyperus rotundus* L., were determined [15]. HPLC analysis revealed the presence of AFs and OTA, with concentrations of up to 31.46 μg/kg and 10.09 μg/kg, respectively. Most of the plants were found to be contaminated with one or both of the mycotoxins tested. The occurrence of aflatoxigenic and ochratoxigenic fungi, as well as the accumulation of AF and OTA, was studied in 80 *Elettaria cardamomum* (L.) Maton. samples collected from different markets in the western region of Saudi Arabia [18]. Using morphological criteria and molecular analysis, the presence of *A. flavus*, *A. parasiticus*, *A. niger*, *A. ochraceus*, *P. citrinum*, and *P. verrucosum* was detected. HPLC showed that total AFs were the predominant mycotoxins, contaminating approximately 67.5% of the *Elettaria cardamomum* (L.) Maton. samples. OTA was found in 47.5% of the samples; notably, 36.3% of them were contaminated with both AFs and OTA. The concentration of AFs and OTA in *Elettaria cardamomum* (L.) Maton. samples ranged from 42.7 to 164.7 μg/kg and from 30.0 to 78.0 μg/kg, respectively. *Matricaria chamomilla* L. samples were collected from local markets and traditional bazaars in Istanbul, Turkey, for the detection of OTA [19]. HPLC-FLD analysis revealed that OTA was present in *Matricaria chamomilla* L. at a low concentration of 0.034 µg/kg. Aromatic and/or medicinal herb samples collected in Spain underwent mycotoxin analysis using ELISA after a cleanup step with multifunctional columns. Among the tested herbs, *Salvia officinalis* L. was found to be significantly contaminated with AFs and OTA, with contamination levels ranging from 23.8 to 25.2 µg/kg for AFs and from 0.11 to 17.3 µg/kg for OTA [23]. A total of 84 medicinal plant and spice samples imported from India, containing *Zingiber officinale* Roscoe, *Foeniculum vulgare* Miller, and *Artemisia absinthium* L., were examined for mold and mycotoxin contamination [26]. *A. flavus*, *A. parasiticus*, *A. niger*, and *P. viridicatum* were most frequently found on the medicinal plant samples. The direct determination of mycotoxins in these samples revealed the presence of AFB_1_ in 17 samples, with concentrations ranging from 10.0 to 160.0 µg/kg, and OTA in 3 samples, with concentrations ranging from 20.0 to 80.0 µg/kg. One study analyzed fifty herbal medicine samples from seven different taxa known for their efficacy in treating liver disorders in India to determine AF contamination [27]. Out of the 50 samples tested, 23 were found to have varying levels of AFs. The highest concentration of AFB_1_ among the contaminated samples was recorded at 2230.0 μg/kg in *Asparagus racemosus* Willd., while the lowest was 280.0 μg/kg in *Phyllanthus emblica* L. Additionally, *A. flavus* was isolated from the tested herbs.

Microscopic techniques are used to determine the quality of herbal drugs [28]. Light microscopy (LM) and scanning electron microscopy (SEM) have been previously used successfully to determine the penetration paths and accumulation of fungal mycelia inside medicinal herbs [29].

As the analytical methodologies for the quantification of AFs and OTs should be fast, selective, simple, accurate, and sensitive, liquid chromatography methods with different detectors (e.g., MS, DAD, and FLD) are the core of AFs and OTs analysis. Gas chromatography (GC) is also utilized for the quantification of AFs and OTs, but it is used to a lesser extent. Gas chromatography with flame ionization detection (GC-FID) and gas chromatography with tandem mass spectrometry (GC-MS/MS) have been used for the screening of AFs and OTs in food samples [30]. Immunoaffinity columns (IACs) are widely used for the cleanup and isolation of AFs and OTs extracted from foods and biological fluids. The columns are prepared by binding antibodies specific to the given AFs and OTs to a specially activated solid-phase support and packing the support suspended in an aqueous buffer solution into a cartridge. The AFs and OTs in the extract or fluid bind to the antibody, impurities are removed with water or an aqueous solution, and then the AFs and OTs are desorbed with a miscible solvent [31].

Three publications have reviewed mycotoxin contamination in medicinal herbs and methods of analysis. In 2013, Santos et al. [32] described mycotoxin contamination in medical and aromatic herbs, its global market significance, processing impacts on contamination in derived foods, and common analytical methods for detecting fungi and mycotoxins. The summaries of four reports [23,24,25,27] mentioned in Table 1 were also included in Santos’s review. Recently, Zhang et al. [12] highlighted analytical techniques for mycotoxin detection in herbal medicines over the past decade, emphasizing sample preparation, conventional chromatographic methods, and advancements in screening assays like ELISAs, lateral flow immunoassays, aptamer-based assays, and cytometric bead arrays. Ałtyn and Twarużek [8] determined the mycotoxicological status of herbal products and highlighted some important challenges associated with the effective monitoring of their safe usage.

In this review, we focus on jujube fruits, lotus seeds, and licorice roots, as these are globally widespread and highly susceptible to mycotoxin contamination. The mechanisms of contamination and the factors affecting mycotoxin accumulation in these herbs, linking their natural properties and maturity to the contamination process, are reviewed.

### 1.1. Aflatoxins (AFs)

AFs, the most common mycotoxins that contaminate crude medicinal plants, are primarily produced by *A. flavus* and *A. parasiticus*. They comprise a group of four major AFs: aflatoxin B_1_ (AFB_1_), aflatoxin B_2_ (AFB_2_), aflatoxin G_1_ (AFG_1_), and aflatoxin G_2_ (AFG_2_) [33]. These are carcinogenic compounds classified as group 1 carcinogens to humans by the International Agency for Research on Cancer (IARC) [34]. AF exposure may be responsible for roughly 25,200–155,000 of the 550,000–600,000 new hepatocellular carcinoma (HCC) cases diagnosed each year worldwide. Further studies have discovered the carcinogenic effects of AFB_1_, which have been attributed mostly to the intermediate metabolite AFB_1_-exo-8,9 epoxide produced from AFB_1_ metabolism by cytochrome P450 enzymes in the liver [13]. Hence, China has established the following relevant standards: the limits for AFB_1_ and total AFs (the combined sum of AFB_1_, AFG_1_, AFB_2_, and AFG_2_) in herbs and decoction pieces are set at 5 and 10 μg/kg, respectively (Chinese Pharmacopoeia, 2015) [35].

### 1.2. Ochratoxins (OTs)

Recently, the presence of OTs in medicinal herbs is considered the most relevant [36]. OTs are toxic secondary metabolites of several strains of fungi, notably *Penicillium verrucosum* in temperate climates and *Aspergillus ochraceus*, *Aspergillus carbonarius*, and *Aspergillus niger* in tropical regions [37]. OTs are closely related to isocoumarin derivatives, linked to an amino acid, L-*β*-phenylalanine, by an amide bond [38]. The optimal production of OTs by *P. verrucosum* in temperate climates occurs at a pH of 6–7, a temperature of 20 °C, and a minimum water activity of 0.86. In tropical regions, the optimum conditions for enhancing the productivity of OTs by ochratoxigenic strains have been reported as a pH range of 3–10, a temperature of 31 °C, and a minimum water activity of 0.8. Additionally, OT production is optimal in the presence of iron, zinc, and copper [29]. Ochratoxigenic strains from the *Penicillium* and *Aspergillus* genera have been isolated from dry licorice root samples [39].

The family of OTs is encompassed by more than 20 different metabolites, among which ochratoxin A (OTA) is the most abundant and most toxic compound [38]. OTA is a mycotoxin known for its carcinogenic (class 2B of IARC), nephrotoxic, teratogenic, and immunotoxic properties, and it has been linked to nephropathy in humans. The Scientific Committee for Food recommended minimizing exposure to OTA as much as possible, establishing a tolerable daily intake of 5.0 ng/kg body weight per day [24].

## 2. AF Contamination of Jujube

*Ziziphus jujuba* Mill. (jujube) is a broadly studied fruit because of its abundant content of phytochemicals, which could encourage a healthy diet [40]. As AF contamination in jujube has become an issue, some papers concerning aflatoxigenic fungi and AF contamination in jujube have arisen. Fifty strains of *A. flavus* were isolated from the preharvest *Ziziphus mauritiana* Lamk. (Indian jujube) in India [41]. An aflatoxigenic strain of *A. flavus* was isolated from the fruits of *Z. Jujuba* Mill. collected in Iraq [20]. Mature fruits of *Z. jujuba* Mill. in Bangkok showed the aflatoxin contamination to be at 2.5–6.1 μg/kg [21]. Two aflatoxigenic *A. flavus* were isolated from *Ziziphus* spp. collected from Zambia markets, which were contaminated in aflatoxin at a low level [16].

Studies have evaluated the relationship between jujube fruit maturation, nutrient concentrations, and AF levels, demonstrating that the maturation process plays a crucial role in the nutritional composition of jujube fruits and their susceptibility to AF contamination [42]. Unsaturated fatty acids, e.g., oleic acid and linolic acid, which are present at a high concentration in mature jujube fruits [43,44], have been shown to inhibit AFB_1_ synthesis, in contrast to saturated fatty acids, e.g., palmitic and stearic acid, and the ratio between unsaturated and saturated fatty acids present in the materials is important in the determination of their susceptibility to AFs’ contamination [45]. Asparagine, which is present at a high concentration in mid-mature jujube fruits but decreases dramatically in mature fruits [46], has been verified to have a significant effect on AFs’ productivity of *A. flavus* [47]. It was found that carbohydrates have a considerable effect on AF production. Soluble sugars that increase by maturity are favorable for AFs’ biosynthesis [48].

**Figure 2:**
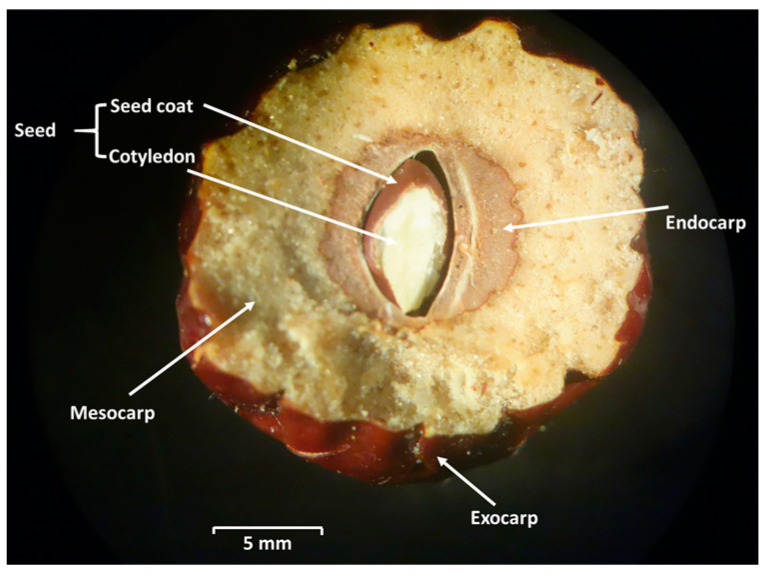
Cross-section of jujube fruit [51].

Jujube fruits have multiplayers (Figure 2); the seeds are surrounded by the pericarp consisting of exocarp, mesocarp, and endocarp [49]. The pericarp is mainly composed of cuticle and epidermal cells. Moreover, the shape, size, and arrangement of epidermal cells differs by stage of maturity. The arrangement changes from compact to relatively loose during maturation. Furthermore, it has been reported that the epidermal cell thickness of jujube fruits decreases with fruit maturity [50]. The endocarp plays a significant role in protecting the seed, which is encased inside. Hardening of the endocarp occurs via secondary cell wall formation and lignification [42]. The secondary lignification of cell walls plays a key role in resistance to various biotic stresses [50].

**Figure 3:**
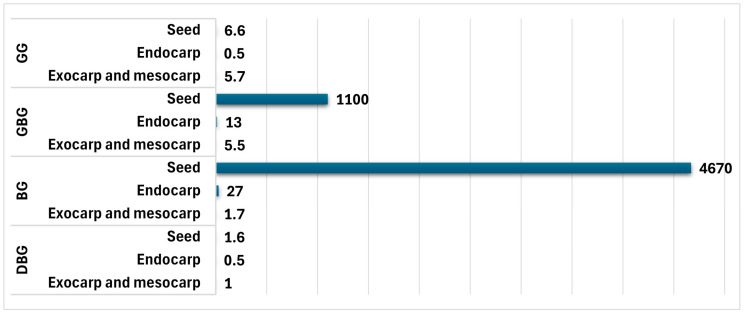
AF concentrations (µg/kg) (total of AFB_1_ and AFB_2_) at each fruit part at different stages of maturity [51].

A study on the susceptibility of the separate parts of jujube fruits at different maturities (the green (GG) of the immature stage, greenish brown (GBG) of the mid-mature stage, brown (BG) of the mid-mature stage, and dark brown (DBG) of the mature stage) to AF contamination demonstrated that the parts of the mid-mature fruits were highly susceptible to AFs compared to the parts of the other mature stages. The seeds are most susceptible to AFs (Figure 3) [51].

**Figure 4:**
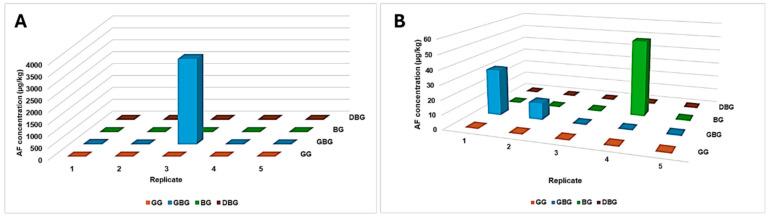
AF concentrations (µg/kg) (total of AFB_1_ and AFB_2_) of intact jujube fruits exposed to *A. flavus* for 15 (**A**) and 30 days (**B**) [42].

When intact fruits of different stages of maturity were inoculated with *A. flavus* spores and incubated for 15 and 30 days [42], the mid-mature fruits had high susceptibility to AF contamination, but large differences in AF concentrations among replicates were observed in both cases of 15 and 30 days of incubation (Figure 4). This fluctuation in the AF concentrations was also observed in the seeds of kernels of GBG fruits which were incubated with *A. flavus* and incubated for 10 days.

The resistance and impermeability of the seed coat part [52], especially testa, to any external substance because of its lignin content, was stated [53]. The seed coat part also limits the activity of physical and biological factors during seed separation process and storage [54,55]. The hilar region (HR) is the only path of water within the seeds because of it being open under the stress of environmental conditions like high humidity and the difference in its structure compared to the testa [56,57].

**Figure 5:**
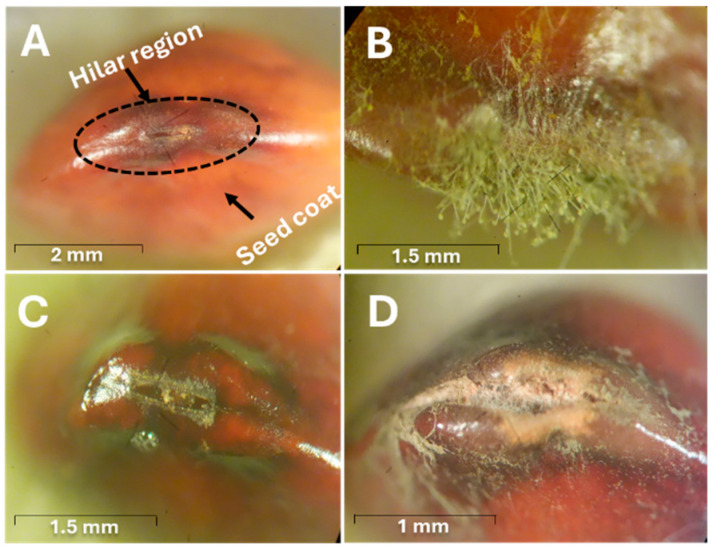
Stereo photomicrographs of HR of jujube seeds. (**A**) Control jujube seed. (**B**) Fungal growth in the HR of jujube seed after 7 days of incubation. (**C**) The HR of jujube seed after 7 days of incubation, after removing the fungal mycelia. (**D**) The HR of jujube seed after 15 days of incubation, after removing the fungal mycelia [51].

A study on the response of HR to fungal mycelial stress [51] showed significant fungal growth at the HR (Figure 5B), which widened after 7 days of incubation (Figure 5C) and was severely affected after 15 days of incubation (Figure 5D).

**Figure 6:**
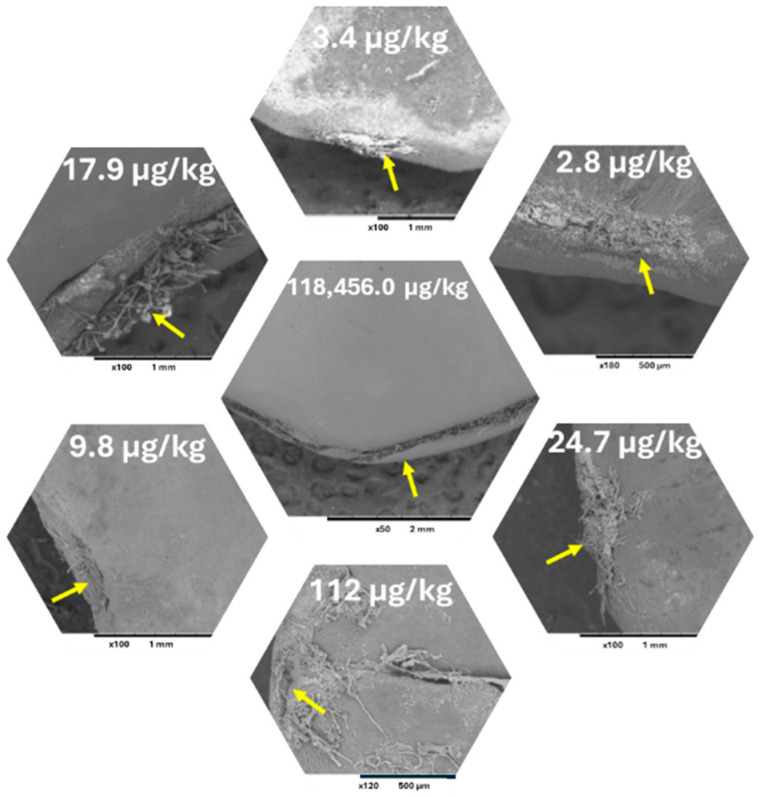
The linkage between the status of the HR of the jujube seeds at the same maturity and AFs accumulation through studying the morphological structure of the HR by SEM and quantifying the AF by HPLC-FLD [51]. Yellow arrows indicate the HR of the jujube seeds.

On the other hand, the positive linkage between the differences in the shape of the HR of the seeds from the same maturity and AF accumulation was revealed (Figure 6).

**Figure 7:**
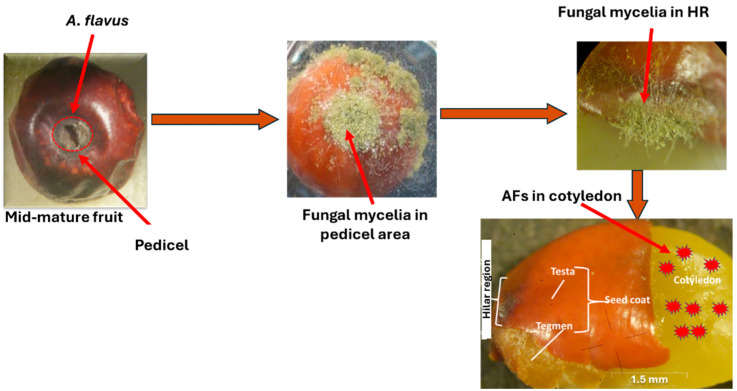
Schematic diagram of the mechanism of AF contamination in intact jujube fruits and their seeds [42,51].

The fungal stress and HR shape were shown as the two crucial factors affecting the fungal mycelial penetration and thus the AF accumulation in jujube seeds. The mechanism could be explained (Figure 7) as fungal mycelia can attack intact jujube by forming infection structures, which are called hyphal differentiations. Accumulation of hyphae forms an infection pad in the contact area between pedicel and fruit, which forms just prior to the penetration of hyphae. The penetrated hyphae accumulated in the HR of the seed. The hyphae involve minor modifications of their morphology, as mentioned by [58]. The resulting increase in pressure within the infection structure further supports the penetration process. Based on the shape of the HR and the width of the hilar fissure, the mycelia could penetrate fast or slowly, which affects the concentrations of the accumulated AFs.

## 3. AF Contamination of Lotus (*Nelumbo nucifera* Gaertn.) Seeds

Lotus (*Nelumbo nucifera* Gaertn.) belongs to the family Nelumbonaceae, which is known for its wide geographical distribution and biological diversity [59]. All parts of the lotus plant are used as food and medicine. Lotus seeds are the resource for both food and medicine, abundant in carbohydrates, lipid, protein, starch, vitamins, minerals, and bioactive compounds which increase with the increase in maturity [17,60]. Lotus seeds are traditionally used to treat various conditions, including nervous disorders, insomnia, high fevers with restlessness, poor digestion, chronic diarrhea, enteritis, tissue inflammation, and cardiovascular diseases such as hypertension and arrhythmia [61].

Lotus seeds are affected by the nature of their components and the external environmental conditions and being susceptible to contamination by aflatoxigenic fungi and subsequent AFs in the preharvest, postharvest, processing, storage, and transportation processes [62,63], especially during rainy seasons, leading to the production and residue of AFs [64]. The analysis of the batches of lotus seed samples gathered from different places over China by UFLC-MS/MS showed that 22% percent of them were contaminated with AFs (45.6–275.6 μg/kg) [17]. Ninety-five percent of the batches of lotus seeds collected from different drug stores or markets in China and analyzed by LC-ESI-MS/MS were contaminated with AFs at levels ranging from 0.02 to 688.4 μg/kg [22].

Lotus seeds are often infected by various fungi during the preharvest stage, potentially leading to AF contamination. *A. flavus* is one such fungus that has been identified in lotus seeds harvested in China [65]. The water gap region in the seed cavity, specifically in the protuberance, is the only permeable area of the lotus seed, allowing for fungal penetration [66]. As the seeds mature, their susceptibility to AF contamination changes, with early immature and mid-mature seeds showing higher AF concentrations, while more mature seeds exhibit lower AF levels [67]. AF concentrations in early immature seeds were higher than those in mid-mature seeds. This indicates that the early stages of development make seeds more vulnerable to fungal contamination and thus AF production.

The permeability of lotus seeds is closely tied to their moisture content. Immature seeds, with higher moisture levels, are more vulnerable to fungal invasion via the water gap region. As the seeds mature, their moisture content diminishes, leading to a natural impermeability against fungal penetration. Environmental humidity plays a significant role in this process, and seeds become increasingly resistant to fungal invasion as they reach full maturity [68,69].

Structural changes in the seed also play a significant role in its defense against fungal infection. The pericarp, which is soft and green in immature seeds, becomes hard and dark brown as the seed matures. This hardened pericarp serves as a physical barrier, preventing fungal penetration. Additionally, exudates from the pericarp can inhibit fungal growth [50,70,71].

**Figure 8:**
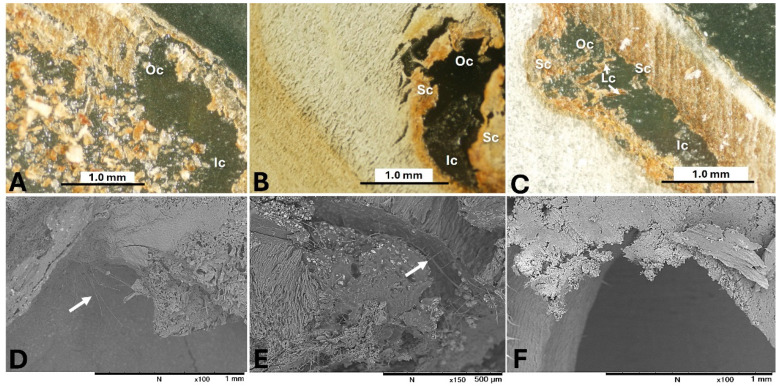
The linkage between the water gap of seeds at different maturities and fungal penetration into the inner parts of the lotus seeds. (**A**–**C**) Light microscope images of the water gap structure of immature, mid-mature, and mature seeds, respectively. (**D**–**F**) Scanning electron microscope (SEM) images of fungal penetration into the inner cavity of the pericarp of immature, mid-mature, and mature seeds, respectively. Abbreviations: Oc, outer cavity of protuberance organ; Ic, inner cavity of protuberance organ; Sc, sclerenchyma cells of wall of protuberance organ; Lc, crystalliferous cells of protuberance organ. White arrows indicate fungal mycelial penetration [67].

As the seed matures, the protuberance (water gap region) undergoes structural changes, with the development of sclerenchyma cells that have thick, lignified secondary walls, and crystalliferous cells. These changes are likely to reduce the permeability of the seed [66] and prevent fungal penetration during the later stages of maturity (Figure 8) [67].

In addition to structural changes, the accumulation of nutrients in the seed, particularly in the embryo, influences its susceptibility to AF contamination. In mid-mature seeds, there is a rapid accumulation of proteins, soluble sugars, amino acids, and fatty acids, which makes the embryo more vulnerable to fungal invasion and subsequent AF contamination. Studies have shown that nutrient accumulation in seeds, especially in embryos, contributes to higher AF contamination [45,48]. This is consistent with findings from similar studies on jujube seeds [51].

Among the different parts of the seed, the embryo contains the highest concentration of AFs, particularly in the mid-mature seeds [67]. This high concentration of AFs in the embryo is linked to the higher nutrient content and the increased susceptibility of the embryo to fungal invasion [68]. Thus, the mechanism of AF contamination in lotus seeds is a complex process influenced by seed maturity, moisture content, structural development, and nutrient accumulation.

**Figure 9:**
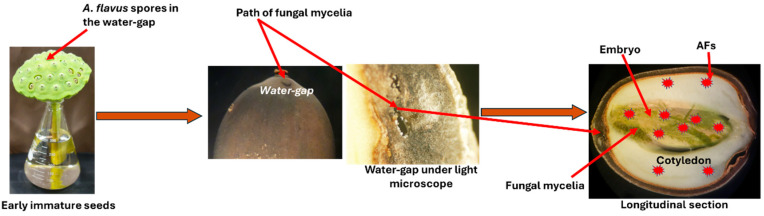
Schematic diagram of the mechanism of AF contamination of the lotus seeds [67].

Figure 9 illustrates the mechanism of fungal mycelial contamination and AF accumulation in the parts of lotus seeds. The fungal mycelia penetrate the seed through the water gap, leading to intensive accumulation in the embryo and cotyledon parts, which significantly produce AFs.

## 4. OTs Contamination of Licorice Roots

Licorice is an herbaceous perennial from the Fabaceae family, classified into three species: *Glycyrrhiza uralensis* Fisch., *Glycyrrhiza inflata* Bat., and *Glycyrrhiza glabra* L. This plant has been utilized in traditional medicine for centuries [72]. The rhizomes and roots are the key medicinal parts of licorice, often used alone or combined with other herbs to treat conditions such as gastric ulcers, sore throat, cough, bronchitis, and arthritis [73,74]. Roots of *Glycyrrhiza* species play a significant role in many traditional Chinese (Kampo) medicinal preparations [75]. However, licorice root is susceptible to fungal contamination and mycotoxin production due to its contact with soil [9,11].

OTA is one of the harmful mycotoxins found in medicinal herbs, including licorice. The European Union enforces strict regulations on OTA levels in licorice, with a maximum of 20.0 µg/kg in licorice root and 80.0 µg/kg in licorice extracts used in confections, as per Commission Regulation (EU) No 105/2010 [36,76]. Over the past decade, a study of more than 71,000 food samples from 29 European countries found the highest OTA levels in plant extract flavorings and essences containing licorice extracts [77]. In addition, distinct studies conducted in Germany and Spain revealed concerning findings: In Germany, 50% of *Glycyrrhiza* sp. root samples and licorice-based sweets showed OTA levels ranging from 0.3 to 216.0 µg/kg [25], while in Spain, all 30 of the tested samples of licorice root and its derived products contained OTA, with levels up to 252.8 µg/kg [24]. Thus, understanding the mechanism of OTA contamination in licorice root is crucial for preventing its spread.

*A. ochraceus* and *A. westerdijkiae*, two important OTA-producing species in *Aspergillus* section Circumdati, are known to contaminate foodstuffs and beverages for human consumption [78]. *A. westerdijkiae*, in particular, is commonly found in both fresh and dried licorice roots cultivated in Xinjiang, China [39]. In these roots, the production of OTA was more dominant than OTB in most tested sections, though certain root parts exhibited higher OTB production [29]. Researchers have studied the ratio of OTA to OTB produced by ochratoxigenic fungi in both fungal cultures and natural materials. Generally, *A. ochraceus* and its taxon, including *A. westerdijkiae*, produce less OTB than OTA in artificial and natural media, with ratios ranging from 1:2 to 1:34. However, under specific culture conditions (e.g., 32% sucrose in a 2% yeast extract solution), the production of OTB and OTA becomes comparable [79,80]. Interestingly, in some instances, such as in 11 of 20 red wine samples from Spain, the production of OTB exceeded that of OTA [81].

Studying the distribution of OTs in different sections of licorice roots is crucial for understanding fungal penetration and OT production. Researchers have used various techniques to examine the spatial distribution of OTs in contaminated materials. An OTA analysis of naturally infected sausage parts using LC-MS/MS found OTA only in the casings, not in the stuffed meat [82]. In artificially inoculated French semi-hard Comté cheese, the highest OTA concentration was near the surface, with minimal production at greater depths after prolonged incubation [83]. Mass spectrometry imaging (MSI) has been used to determine mycotoxin distribution in plant tissues [84]. MALDI-MSI analysis of OTA contamination in vegetable foodstuffs showed OTA co-localizing with visible fungal spoilage [85]. In line with these studies, a study by [29] using desorption electrospray ionization tandem mass spectrometry (DESI-MS/MS) demonstrated that OTs accumulate in the cut and damaged areas of licorice roots, with only a slight concentration observed in the deeper sections, despite fungal penetration. This finding was confirmed through microscopic observations using light microscopy (LM) and scanning electron microscopy (SEM).

Fungal penetration into licorice roots is complex due to strong defense mechanisms, including physical and chemical barriers in the cork layer. These defenses involve antifungal compounds like glabridin, suberization, lignification, and gum accumulation [86,87]. These mechanisms help prevent infection by ochratoxigenic fungal strains, as the cork layer blocks fungal penetration [29]. However, fungal pathogens exploit weak spots such as cuts, wounds, and stomata to access nutrients, forming infection structures that apply pressure to breach cell walls and enter root tissue [58]. The fungal hyphae then infiltrate the roots, producing spores, which are visible under light and electron microscopy [29].

**Figure 10:**
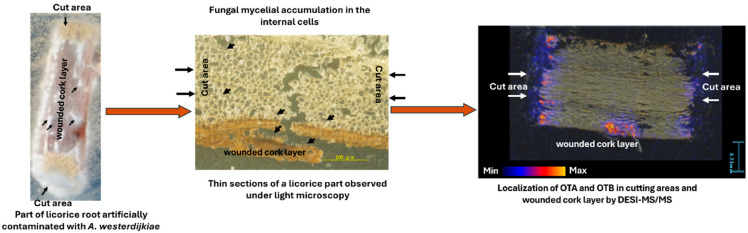
The mechanism of fungal mycelia and OT contamination of licorice [29].

Figure 10 illustrates the mechanism of fungal and OT contamination in licorice roots based on the findings of Elamin et al. [29]. Fungal mycelia accumulated in the cutting areas and wounders in the licorice parts, forming infection pads. The fungal mycelia penetrated the licorice and accumulated within the licorice’s internal cells. The DESI-MS/MS revealed high OT concentrations in cut root areas and damaged cork layers, with fewer OTs present in the deeper root and cork layers, suggesting that the fungus primarily targets and contaminates the outer sections. The cork layer, due to its structure and high lignin content, is more resistant to fungal penetration [88]. LM imaging showed near-complete fungal penetration into the root, causing some cell walls to collapse or degrade. These observations imply that the areas of fungal growth and OT accumulation do not always coincide.

## 5. Conclusions

AFs and OTA pose significant health risks, particularly when present in medicinal herbs that are widely used across all societies. The interaction between the natural defenses of medicinal herbs, such as jujube, lotus, and licorice, as well as fungal pathogenicity, is a complex and multifaceted relationship. This relationship involves the chemical composition and physical characteristics of the herbs, which vary with the stages of maturity, and individual differences among plants of the same growth stage.

Through this review, the mechanisms of AF and OT contamination in the studied medicinal herbs were described. It became evident that despite the protective layers surrounding lotus and jujube seeds, *A. flavus* can develop a mechanism to penetrate these layers. Seeds in the mid-mature stages are more susceptible to fungal penetration and AF accumulation due to their rich chemical profile, which is necessary for AF production. Additionally, the structure of the HR, the only path to the seeds, remains penetrable, unlike in the mature stages. It also became clear that the individual differences between the morphological structures of the seed HR at the same maturity stage affect the degree of permeability and are therefore crucial factors in accelerating or slowing down the mechanism of AF accumulation.

In licorice, the cork layer effectively limited fungal penetration. It became clear that the distribution of OTs was consistently adjacent to the cut surface, and despite mycelial penetration into the inner structure, OT production in the deeper parts of licorice remained limited.

## Figures and Tables

**Table 1:** AFs and OT contamination in medicinal herbs.

Herb	Country	Mycotoxin	Concentration (μg/kg)	Fungi	Year	Ref.
Plant food supplements: 1. *Centella Asiatica* (L.) Urb., 2. *Hippocratea indica* Willd., *Piper nigrum* L., *Trachyspermum ammi Trachyspermum ammi* (L.) Sprague, *Quercus infectoria* Olivier, *Labisia pumillia* (Blume) Fern.-Vill., 3. *Phoenix dactylifera* L., *Nigella sativa* L., *Piper betle* L., *Crocus sativus* L., and 4. *Punica granatum* L., *Zingiber officinale* Roscoe, *Quercus infectoria* Olivier. *Elephantopus scaber* L., *Plectranthus* L., *Labisia pumila* (Blume) Fern.-Vill.	Malaysia	AFB_1_	5.905–13.941	-	2023	[13]
typhoid herbs: Bark of *Enantia chlorantha* Oliv., *Sarcocephalus latifolium* (Sm.) E.A. Bruce, *Garcinia kola* Heckel and *Cocos nucifera* L.	Nigeria	Total AFs	≤7600.0	*A. flavus*	2023	[14]
*Codonopsis* radix, *Scutellariae* radix (the dried roots of *Scutellaria baicalensis* Georgi) and *Tremella fuciformis* Berk.	China	AFB_1_ OTA	0.9–3.05 37.0–515.0	*A. flavus* and *Penicillium* spp.	2020	[9]
*Harmbstaedti aodorata* (Burch.) T. Cooke, *Vachellia karroo* (Hayne) Banfi & Galasso, and *Cyperus rotundus* L.	South Africa	Total AFs OTA	2.0–31.46 2.4–10.09	-	2020	[15]
*Ziziphus* spp.	Zambia	Total AFs	ND-24.4	*A. parasiticus* and *A. flavus*	2019	[16]
*Nelumbo nucifera* Gaertn.	China	Total AFs	45.6–275.6	-	2019	[17]
*Elettaria cardamomum* (L.) Maton.	Saudi Arabia	Total AFs OTA	42.0–164.7 30.0–78.0	*A. flavus*, *A. parasiticus*, *A. niger*, *A. ochraceus* and *P. verrucosum*	2018	[18]
*Matricaria chamomilla* L.	Turkey	OTA	0.034 (below LOD)	-	2018	[19]
*Ziziphus jujuba* Mill.	Iraq	Total AFs	144.0	*A. flavus*	2017	[20]
*Ziziphus jujuba* Mill.	Thailand	Total AFs	2.5–6.1	*-*	2017	[21]
*Nelumbo nucifera* Gaertn.	China	Total AFs	ND- 688.4	*-*	2013	[22]
*Salvia officinalis* L.	Spain	Total AFs OTA	23.8–25.2 0.1.1–17.3	*Aspergillus* spp., *Penicillium* spp.	2009	[23]
*Glycyrrhiza* sp.	Spain	OTA	≤152.8	*-*	2007	[24]
*Glycyrrhiza* sp.	Germany	OTA	0.3–216.0	-	2000	[25]
*Zingiber officinale* Rosoe, *Foeniculum vulgare* Miller, and *Artemisia absinthium* L.	India	AFB_1_ OTA	25.0–160.0 20.0–80.0	*A. flavus*, *A. parasiticus*, *A. niger*, and *P. viridicatum*	1998	[26]
*Phyllanthus emblica* L., and *Asparagus racemosus* Willd.	India	AFB_1_	280.0–2230.0	*A. flavus*	1993	[27]

## Data Availability

No new data were created or analyzed in this study.
